# Residual root fate after aortic surgery in bicuspid aortic valve with right‐to‐left fusion: A comparative risk analysis

**DOI:** 10.1111/jocs.15585

**Published:** 2021-05-07

**Authors:** Nicola Pradegan, Danila Azzolina, Dario Gregori, Gianmarco Randazzo, Sara Frasson, Gino Gerosa

**Affiliations:** ^1^ Cardiac Surgery Unit, Department of Cardiac, Thoracic, Vascular Sciences and Public Health Padua University Hospital Padova Italy; ^2^ Biostatistics Unit, Department of Cardiac, Thoracic, Vascular Sciences and Public Health University of Padova Padova Italy

**Keywords:** aorta and great vessels, aortic root, aortic surgery, bicuspid aortic valve

## Abstract

**Background and Aim:**

Although bicuspid aortic valve (BAV) anatomy might influence aortic aneurysm development, BAV‐related root involvement still lacks standardized surgical management. We aimed to evaluate late clinical outcomes and risk factors for root dilation after proximal aortic replacement in patients with BAV and right–left fusion (RL‐BAV).

**Methods:**

Clinical and echocardiographic data of all patients with intraoperative RL‐BAV who underwent ascending aortic replacement with or without noncoronary sinus (NCS) replacement (Groups 1 and 2, respectively) between 1999 and 2017, were retrospectively revised. A multivariable analysis assessed hazard factors for root dilation during follow‐up (FU).

**Results:**

Of 206 surgeries performed (M 81%; age: 57 ± 13 years, EuroSCORE II: 2.7 ± 1.9%), 79 (38%) required NCS replacement. One hundred fifty‐seven patients (76%) underwent aortic valve replacement (with aortic regurgitation predominating in Group 1, *p* = .04). The preoperative aortic root was larger in patients requiring NCS replacement (43.3 ± 5.1 vs. 39.2 ± 4.8 mm, *p* < .001). At a median FU time of 7 years (interquartile range: 4–10), no residual root dissections occurred, and only two patients (belonging to Group 2) required redo root surgery. Preoperative mild aortic regurgitation and aortic root diameter >35 mm at discharge were risk factors for root dilation >40 mm at FU (*p* = .02). Aortic root did not dilate over time, irrespective of NCS replacement (*p* = .06).

**Conclusions:**

Aortic root in patients with RL‐BAV undergoing ascending aortic replacement (±NCS replacement) does not significantly dilate over time, even if patients with preoperative aortic regurgitation and postoperative root more than 35 mm might require more surveillance.

## 1 INTRODUCTION

Bicuspid aortic valve (BAV) is the most common congenital heart defect,^1^ it is associated with aortic valve disease and aneurysm formation, with an increased risk of developing acute aortic dissection. Aortic dilation involves either the arch and the proximal aorta (aortic root and/or ascending aorta) according to BAV anatomy. BAV with right‐to‐left fusion pattern (RL‐BAV) is the most common anatomic pattern,^1^ and it is frequently related to aortic root enlargement. Even if genetic and hemodynamic factors contribute to the heterogeneity of BAV aortopathy, there is no consensus about their degree of involvement, interaction, and implication for surgical management. Therefore, even if the most recent guidelines agree on thresholds for aortic repair^2^ and many studies have recognized that aortic root does not dilate over time when not replaced,^3^, ^4^ it is still not well established which is the best surgical strategy and when a specific technique should be preferred above others in root surgery, especially regarding BAV anatomy. We aimed to evaluate late clinical outcomes and risk factors for root dilation after proximal aortic replacement in patients with RL‐BAV.

## 2 MATERIAL AND METHODS

### 2.1 Patient selection

**Figure 1:**
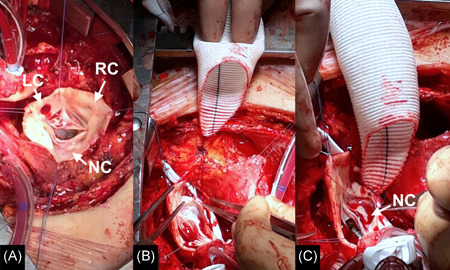
Surgical image of the noncoronary sinus replacement technique: first, the bicuspid valve is exposed and its anatomy is defined (LC, left‐coronary cusp; NC, noncoronary cusp; RC, right coronary cusp) (A); the Dacron prosthesis is prepared and sutured starting from the noncoronary sinus (B and C)

**Figure 2:**
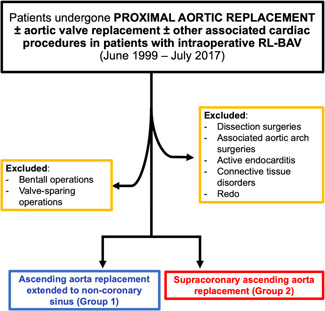
CONSORT flow diagram showing inclusion and exclusion criteria of this study

All subjects who underwent ascending aortic surgery between June 1999 and July 2017 at the Cardiac Surgery Department of the Padua University Hospital were identified through the operative reports database. We selected all those cases with an intraoperative finding of RL‐BAV (±aortic valve disease with surgical indication) and who underwent: ascending aortic replacement extended to the noncoronary sinus (NCS) (Group 1), or supracoronary ascending aortic replacement (Group 2). All operations were performed by means of full sternotomy. Technically, in Group 1 patients, NCS was replaced molding the dacron prosthesis used for ascending aortic replacement (Figure 1A–C). NCS was replaced in patients with root diameters more than 45 mm and indications for aortic valve surgery, or in patients with root diameters less than 45 mm, associated aortic valve disease, and a thin and asymmetrically dilated root. Current guidelines for aortic surgery were used.^2^ Patients who underwent a David (reimplantation or remodeling technique) or a Bentall operation were excluded. Emergent surgery (e.g., dissection), associated aortic arch surgery, active endocarditis, connective tissue disorders (e.g., Marfan syndrome, Loeys–Dietz syndrome, etc.), and redo operations were also exclusion criteria (Figure 2).

### 2.2 Data collection

Perioperative, intraoperative, and postoperative clinical data were retrieved from our institutional database. Patients were contacted via phone or electronically to assess their clinical status between September 2017 and September 2018. Imaging data were collected from all available echocardiography reports at baseline, at discharge, and during follow‐up (FU). Echocardiographic data at FU were obtained between September 2017 and September 2018 directly from the patient via phone, electronically, or by contacting patients' community cardiologists. We only accepted the last echocardiogram (not older than 1 year since the FU date) for each patient. All available aortic root and ascending aortic dimension data were collected and analyzed. The Research Ethics Board of the University Health Network approved the study. Each patient's informed consent was obtained.

### 2.3 Statistical analysis

Descriptive clinical characteristics were reported as median (I and III quartiles) or mean ± standard deviation for continuous variables, and percentages (absolute numbers) for categorical variables. Wilcoxon–Kruskal–Wallis test was performed for continuous variables and Pearson *χ* ^2^ test for categorical ones.

A cumulative incidence curve was represented for cardiac reoperations outcome, accounting for the competitive risk together with a competitive risk regression Fine–Gray model.

A multivariable Cox regression cause‐specific hazard model was estimated to evaluate risk factors of aortic root dilation more than 40 mm at FU. The covariates included in the model were selected according to clinical judgment (gender, age, preoperative aortic regurgitation, preoperative aortic stenosis, NCS replacement, preoperative root diameter, aortic valve replacement). The proportionality of hazard was evaluated using proportional hazards tests and diagnostics based on weighted residuals.

A longitudinal linear mixed‐effects model was used to analyze all the available echocardiographic data at FU between the two groups. An interaction term between time and treatment was considered in the model.

A *p* < .05 was considered statistically significant. Computations have been performed using the R 3.5.2 System and rms package.

## 3 RESULTS

### 3.1 Baseline characteristics

Between June 1999 and July 2017, 206 patients with intraoperative RL‐BAV underwent ascending aortic surgery at our center and met our inclusion criteria. The preoperative characteristics are shown in Table 1. Among this cohort, 79 patients underwent ascending aortic replacement extended to the NCS (Group 1), and 127 patients underwent supracoronary ascending aortic surgery (Group 2). Patients were predominantly male (>75%), and did not significantly differ in terms of age and EuroSCORE II risk among the two groups (*p* = .52 and .78, respectively). Group 1 and Group 2 required associated aortic valve replacement in more than three‐fourth of patients (75% and 77%, respectively); predominant indications were aortic regurgitation in Group 1, and aortic stenosis in Group 2 (*p* < .01). Group 1 showed a low rate of postoperative reoperations for bleeding (*n* = 2, 3%) with no significant difference compared to Group 2 (*p* = .57). There were no differences between the two groups in terms of hospital stay and in‐hospital mortality (*p* = .44 and .43, respectively). Intraoperative and postoperative outcomes are summarized in Table 2.

**Table 1:** Preoperative clinical characteristics of patients

Variable	Group 1 (*n* = 79)	Group 2 (*n* = 127)	*p* Value
Age (years)	55 (48–67)	59 (48–66)	.52
Male sex	71 (91%)	96 (76%)	.008
Body mass index (kg/m^2^)	25.6 (23.7–28.1)	25.9 (24.1–28.7)	.39
Hypertension	46 (58%)	76 (60%)	.82
Diabetes	11 (14%)	7 (6%)	.04
Peripheral vasculopathy	3 (4%)	3 (2%)	.55
Chronic obstructive pulmonary disease	0	4 (3%)	.11
Chronic liver disease	8 (10%)	6 (5%)	.14
New York Heart Association Class			.003
I	60 (76%)	73 (57%)	
II	18 (23%)	40 (31%)	
III	0	14 (11%)	
IV	1 (1%)	0	
Euroscore II (%)	2.0 (1.6–3.0)	2.2 (1.6–3.3)	.78
Aortic valve disease			
Stenosis ≥ moderate	29 (36%)	73 (58%)	.004
Regurgitation ≥ moderate	39 (49%)	52 (41%)	.04
Aortic root diameter (mm)	43.0 (40.5–46.0)	39.0 (35.0–42.0)	<.001
Ascending aorta diameter (mm)	50.0 (46.0–52.0)	50.0 (46.5–52.0)	.76
Left ventricular ejection fraction (%)	62 (57–66)	60 (55–65)	.34

*Note*: *p* Values refer to an overall difference among the two groups.

**Table 2:** Intraoperative and early postoperative outcomes

Variable	Group 1 (*n* = 79)	Group 2 (*n* = 127)	*p* Value
Isolated ascending aorta replacement	14 (18%)	23 (18%)	.44
Associated cardiac surgery			
AVR	50 (64%)	87 (68%)	
AVR + CABG	9 (11%)	11 (9%)	
Aortic valve repair	5 (6%)	2 (2%)	
Mitral valve repair/replacement	1 (1%)	4 (3%)	
Size of tubular prosthesis (mm)	30 (30–32)	30 (30–32)	.12
Size of aortic prosthesis (mm)	25 (23–25)	24 (23–25)	.02
Type of prosthesis			.69
Bioprosthesis	39 (64%)	62 (63%)	
Mechanical prosthesis	20 (36%)	36 (37%)	
Cardiopulmonary bypass time (min)	175 (145–203)	155 (128–190)	.02
Cross‐clamp time (min)	140 (120–163)	120 (100–145)	.003
RBC units transfused			.32
0	54 (68%)	83 (65%)	
1–2	19 (24%)	35 (28%)	
>2	6 (8%)	9 (7%)	
Plasma units transfused			.69
0	56 (71%)	89 (70%)	
1–3	14 (18%)	29 (23%)	
>3	9 (11%)	9 (7%)	
Reoperation for bleeding	2 (3%)	5 (4%)	.57
Postoperative myocardial infarction	0	0	
Stroke or transient ischemic attack	3 (4%)	0	
Postoperative atrial fibrillation	4 (5%)	21 (17%)	
Intensive care unit stay (days)	1 (1–2)	1 (1–1)	.68
Hospital stay (days)	8 (7–11)	8 (7–12)	.44
In‐hospital mortality	0	1 (1%)	.43
Discharge aortic root diameter (mm)	34.0 (32.0–37.0)	33.0 (30.0–36.0)	.06

*Note*: *p* Values refer to an overall difference among the two groups.

Abbreviations: AVR, aortic valve replacement; CABG, coronary artery bypass graft; ICU, intensive care unit; RBC, red blood cells.

### 3.2 FU outcomes

**Figure 3:**
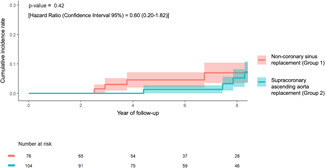
Cumulative incidence curve for cardiac reoperations at follow‐up between Group 1 (ascending aortic replacement extended to the noncoronary sinus, red) and Group 2 (supracoronary ascending aortic replacement, green), with patients at risk at follow‐up. There was not a significant difference between the two cohorts (*p* = .42)

FU was completed for 194 (94%) patients. The median (interquartile range [IQR]) FU time was 7 (4–10) years (maximum 14 years). Although patients who underwent NCS replacement presented higher overall mortality (*n* = 14, 18%), only four patients died for cardiovascular disease (two due to endocarditis, one due to heart failure secondary to aortic bioprosthesis failure, one due to dilatative cardiomyopathy). Asymptomatic patients (according to New York Heart Association Class) were prevalent, with a low rate of neurologic complications at FU (Table 3). During the FU period, 11 patients required cardiac reoperations (Group 1 = 6, Group 2 = 5), especially for aortic bioprosthesis deterioration. More details are shown in Table 4. Only two patients belonging to Group 2 required a root replacement due to aortic root enlargement (none in Group 1). The cumulative incidence rate for cardiac reoperations did not find any significant difference between the two groups (*p* = .42) (Figure 3).

**Table 3:** Follow‐up details

Variable	Group 1 (*n* = 78)	Group 2 (*n* = 116)	*p* Value
Overall mortality	14 (18%)	8 (7%)	.01
New York Heart Association Class			.99
I	68 (87%)	107 (92%)	
II	10 (13%)	9 (8%)	
>III	0	0	
Complications			.07
Stroke or transient ischemic attack	1	2	
Aortic dissection	0	0	
Endocarditis	2	4	
Cardiac reoperations	6	3	
Follow‐up aortic root diameter (mm)	40.0 (35.0–44.0)	37.0 (33.0–39.00)	.01

*Note*: *p* Values refer to an overall difference among the two groups.

**Table 4:** Details of cardiovascular reoperations

Patient	Group	Procedure	Years postoperative	Indication for surgery
1	1	Redo AVR	12	Aortic prosthesis dysfunction
2	1	Redo AVR	2	Aortic prosthesis dysfunction
3	1	Redo AVR	12	Aortic valve stenosis
4	1	MVR + CABG + PFO repair	4	Mitral regurgitation, CAD, PFO
5	1	TAVR valve‐in‐valve	10	Aortic prosthesis deterioration
6	1	AVR + MVR + CABG	6	Aortic insufficiency, mitral regurgitation, CAD
7	2	Redo AVR + MVR	11	Aortic prosthesis dysfunction
8	2	TAVR valve‐in‐valve	7	Aortic prosthesis dysfunction
9	2	CABG + Bentall procedure	4	CAD, aortic root dilation (50 mm)
10	2	TAVR valve‐in‐valve	8	Aortic prosthesis dysfunction
11	2	Bentall procedure	9	Aortic root dilation (48 mm)

Abbreviations: AVR, aortic valve replacement; CABG, coronary artery bypass graft; CAD, coronary artery disease; MVR, mitral valve replacement; PFO, patent foramen ovale; TAVR, transcatheter aortic valve replacement.

### 3.3 Aortic root echocardiographic analysis

**Figure 4:**
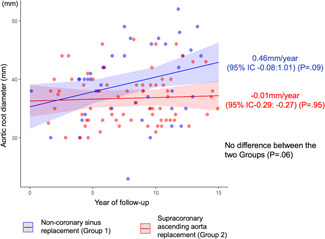
Echocardiographic aortic root diameter over follow‐up time in Group 1 (ascending aortic replacement extended to the noncoronary sinus, blue color) and Group 2 (supracoronary ascending aortic replacement, red color). Dots represent the observed root diameters at follow‐up, lines are an estimation of the residual root fate after surgery. Aortic root diameter slowly increases by 0.46 mm/year (95% confidence interval [CI]: −0.08 to 1.01) (*p* = .09) in Group 1, and by −0.01 mm/year (95% CI: −0.29 to −0.27) (*p* = .95) in Group 2. According to a linear mixed model, the estimated progression rate in Group 1 versus Group 2 is not significant (*p* = .06)

One hundred forty‐three (74%) FU echocardiograms were collected within the study period (with a median length of FU echo studies = 7 years, IQR: 4–9). When analyzing aortic root diameters, we found that preoperative values in patients who underwent NCS replacement were significantly larger than patients who underwent supracoronary ascending aorta replacement (median, IQR = 43.0 mm, 40.5–46.0 vs. 39.0 mm, 35.0–42.0, *p* < .001). After surgery, there was a significant reduction in root diameters at discharge (*p* < .0001), with similar values between Groups 1 and 2 (*p* = .06). Statistical analysis of all available echocardiographic imaging reports revealed that aortic root diameter slowly increased during FU by 0.46 mm/year (95% confidence interval: −0.08 to 1.01) (*p* = .09) in patients with NCS replacement, and by −0.01 mm/year (95% confidence limits: −0.29 to −0.27) (*p* = .95) in patients with no NCS intervention. The differential progression rate between the two groups was not significant (*p* = .06) (Figure 4). Multivariable analysis of risk factors for root dilation at FU is summarized in Table 5 and shows that preoperative aortic regurgitation and dilated root at discharge are the only factors related to increased risk of dilation at FU (*p* = .02).

**Table 5:** Multivariable Cox regression analysis of risk factors for aortic root dilation more than 40 mm at follow‐up

	HR	CI 95%	*p* Value
Male sex	1.71	0.38–7.77	.49
Age (48–66 years)	1.82	0.93–3.59	.08
Preop aortic regurgitation ≥ moderate	4.39	1.23–15.67	.02
Preop aortic stenosis ≥ moderate	0.5	0.09–2.71	.42
Noncoronary sinus replacement	2.1	0.93–4.77	.08
Aortic root diameter at discharge > 35 mm	3.45	1.26–9.39	.02
No aortic valve replacement	3.64	0.98–13.46	.05

*Note*: The hazard ratio (HR) with a 95% confidence interval (CI) has been reported. The interquartile effect has been expressed for continuous variables.

## 4 DISCUSSION

BAV, the most common congenital heart defect in adults,^5^, ^6^ is usually complicated by the development of aortic stenosis or regurgitation. However, aortic dilation from the aortic root to the aortic arch (bicuspid aortopathy) is also present in approximately 50% of affected people.^1^ Hemodynamic and genetic factors seem to play a combined role in the development of BAV aortopathy.^7^, ^8^ Even if current guidelines clearly establish limit thresholds for aortic replacement in BAV patients,^2^ aortic root management still remains unresolved. Many studies have advocated supracoronary ascending aortic replacement as a treatment (preserving the intact moderately dilated sinus segment and coronary ostia),^9^, ^10^ whereas others suggest removal of the sinus segment by means of a valve‐sparing procedure or a Bentall operation.^11^ However, there is limited comparative analysis regarding the stability of the residual root after aortic surgery (either with or without aortic valve replacement) according to BAV morphology. We selectively analyzed RL‐BAV patients because the most common fusion pattern clinically encountered involves the right and left cusps.^1^ Even if RL‐BAV pattern is most frequently related to type 1 BAV aortopathy (which preferentially includes patients >50 years of age, with associated aortic stenosis and different degrees of aortic root dilation),^7^, ^12^ BAV regurgitation is typically associated with root phenotype.^13^, ^14^ Our results confirm this evidence: in fact, patients with a more dilated aortic root and who underwent an associated partial root procedure (Group 1) suffered more frequently of moderate‐severe aortic regurgitation rather than stenosis. The more “aggressive” approach of replacing NCS arises from the intraoperative evaluation of different factors. Similarly to other experiences,^15^, ^16^ beyond aortic root size, we usually consider sinuses of Valsalva (SOV) replacement if the aortic wall looks thinner than usual. Besides this, we selectively replace NCS if we find an asymmetric dilation of the NCS rather than circumferential root dilation. Even if these data are similar to a smaller cohort by Caynak et al.^17^ (which analyzed 99 patients, and compared surgical results between those who underwent Bentall and NCS replacement), we still think that the Bentall operation is a complex procedure even in experienced hands: CPB times are usually longer, intraoperative bleeding and kinking of the graft can be difficult to manage, and late problems (e.g., aneurysms of the arterial buttons, periaortic fistulas) can occur.^18^, ^19^ On the other hand, when considering rates of aortic root reoperation or root dissection in patients undergoing selective NCS replacement, the risk of these complications is extremely rare (as shown in this paper and as previously reported by other groups^20^, ^21^, ^22^).

In the last few years, there has been an increasing interest in investigating the fate of aortic root after ascending aorta replacement and/or aortic valve replacement, both in BAV and in the tricuspid aortic valve. Among different studies,^3^, ^4^, ^9^ Milewski et al.^3^ and Hui et al.^4^ recently confirmed that both in patients who underwent supracoronary ascending aortic replacement or those who received associated NCS replacement, the residual aortic root did not significantly dilate over time. Particularly, Milewski et al.^3^ stated that SOV dimensions remain stable over long‐term FU, irrespective of valvular morphology and pathology. Our experience strengthened this previous evidence, confirming that the aortic root is significantly remodeled in terms of diameter, either when NCS is replaced or not in the context of ascending aortic replacement. Besides this, over long‐term FU the root (regardless of the surgery performed) dilates at a rate that is not of clinical relevance. Similar to Hui et al.,^16^ we found a similar progression of sinusal diameter after surgery, with no difference either replacing or not NCS. This stability might be explained by the different embryogenic origins of the aortic root (neural crest origin or secondary heart field^23^, ^24^). Similarly to these previous studies,^3^, ^4^ we also showed a reduction of root diameter in patients undergoing supracoronary ascending aorta replacement: this might be explained by the fact that when the vascular prosthesis is anastomosed to the sinotubular junction (STJ), the mismatch between STJ and the prosthesis might indirectly reduce the root diameter.

Differently from previous references, our multivariable analysis found that larger diameters at discharge and preoperative aortic regurgitation were significant risk factors of aortic root dilation at FU. As aortic regurgitation is strongly correlated to root phenotype, we hypothesize that there are some unknown genetic features of the aortic root when associated with regurgitation which make it more prone to enlargement.^25^ Patients who did not undergo AVR were also at increased risk of dilation, despite a not significant *p* value. We think that the prosthetic stent might stabilize the annulus and the root; therefore, BAV, even if continent and not stenotic, produces abnormal flows and shear stress into the sinusal portion, and this might explain a higher predisposition to dilation.^8^, ^26^, ^27^

This study has the following limitations. First, it is a retrospective study, and surgeries were performed by several surgeons working at our center. Aside from current guidelines, the decision to replace NCS or not was mostly based on surgeon preference. Clinical and echocardiographic FU was not 100% complete, as well as median clinical FU is short. The three groups show a disproportion in the total amount of patients included, and larger patients groups would be mandatory. Aortic measurements were performed by different cardiologists in different centers with different machines; a systematic evaluation of all the available images by a single examiner (blinded to patients group) would have reduced the error margin.

In conclusion, our study shows that aortic root in patients with RL‐BAV who undergo proximal aortic replacement does not significantly dilate over time (irrespective of SNC replacement). Patients with preoperative aortic regurgitation and postoperative residual aortic root dilation (>35 mm) seem more prone to dilation at FU and might require surveillance. However, surgical reoperations for aortic root dissection or dilation are extremely rare at FU, so that full replacement of the aortic root when only mildly dilated still does not appear justified. NCS replacement in the asymmetrically dilated root might be easier and less risky (Figure 4).

## CONFLICT OF INTERESTS

The authors declare that there are no conflict of interests.

## AUTHOR CONTRIBUTIONS

**Nicola Pradegan**: Data analysis/interpretation, drafting article, critical revision of the article. **Danila Azzolina**: Statistics. **Dario Gregori**: Statistics, critical revision of the article. **Gianmarco Randazzo**: Data collection. **Sara Frasson**: Data collection. **Gino Gerosa**: Concept/design, critical revision of the article, approval of the article.
